# Pathological Crosstalk Between Oxidized LDL and ER Stress in Human Diseases: A Comprehensive Review

**DOI:** 10.3389/fcell.2021.674103

**Published:** 2021-05-26

**Authors:** Divya Saro Varghese, Bassam R. Ali

**Affiliations:** ^1^Department of Genetics and Genomics, College of Medicine and Health Sciences, United Arab Emirates University, Al Ain, United Arab Emirates; ^2^Zayed Bin Sultan Center for Health Sciences, United Arab Emirates University, Al Ain, United Arab Emirates

**Keywords:** ER stress sensors, UPR arm, human disease, HDL, oxidized LDL, therapy

## Abstract

The oxidative modification of the major cholesterol carrying lipoprotein, oxLDL, is a biomarker as well as a pathological factor in cardiovascular diseases (CVD), type 2 diabetes mellitus (T2DM), obesity and other metabolic diseases. Perturbed cellular homeostasis due to physiological, pathological and pharmacological factors hinder the proper functioning of the endoplasmic reticulum (ER), which is the major hub for protein folding and processing, lipid biosynthesis and calcium storage, thereby leading to ER stress. The cellular response to ER stress is marked by a defensive mechanism called unfolded protein response (UPR), wherein the cell adapts strategies that favor survival. Under conditions of excessive ER stress, when the survival mechanisms fail to restore balance, UPR switches to apoptosis and eliminates the defective cells. ER stress is a major hallmark in metabolic syndromes such as diabetes, non-alcoholic fatty liver disease (NAFLD), neurological and cardiovascular diseases. Though the pathological link between oxLDL and ER stress in cardiovascular diseases is well-documented, its involvement in other diseases is still largely unexplored. This review provides a deep insight into the common mechanisms in the pathogenicity of diseases involving oxLDL and ER stress as key players. In addition, the potential therapeutic intervention of the targets implicated in the pathogenic processes are also explored.

## Introduction

Over the past few decades, dysregulated cholesterol and lipid homeostasis have contributed to the worldwide rapid progression of lifestyle diseases, collectively termed the metabolic syndrome (MetS) or syndrome X. It is estimated that over a billion people are affected by this global epidemic (Saklayen, 2018). This new non-communicable disease is characterized by a combination of dyslipidemia, cardiometabolic disorders, hypertension, insulin resistance, type II diabetes mellitus (T2DM), obesity, non-alcoholic fatty liver disease (NAFD) and non-alcoholic steatohepatitis (NASH) (Amihăesei and Chelaru, 2014). The anomalies manifested in these systemic diseases primarily target the cardiovascular system, liver, pancreas and kidney that eventually lead to chronic and lethal effects on the individual. Oxidized low-density lipoprotein (oxLDL) plays a pivotal role in dyslipidemia associated MetS (Berneis and Krauss, 2002). In this review, we explore human metabolic diseases involving the pathological interaction between oxLDL and Endoplasmic Reticulum (ER) stress that culminate in morbidity. Further, it would provide a better insight on identifying common targets and biomarkers for improved prognosis, diagnosis and therapy.

## Lipids: An Overview

Lipids are comprised of triglycerides, phospholipids, sterols, and fat-soluble vitamins; of which triglycerides and cholesterol are the major sources of fat found in the blood. Lipid metabolism is the collective process of synthesis and degradation of lipids and their derivatives within or outside- cellular compartments. The composition of lipids varies from cell to cell, depending on the structure and function of the organelle. In addition to contributing to the structural organization of the cell by providing building blocks that maintain membrane plasticity, lipids play dynamic roles in membrane trafficking and signal transduction (Grouleff et al., 2015; Santos and Preta, 2018). By reason of its structural as well as functional relevance, lipid metabolism is a tightly regulated process that is influenced by the balance between endogenous or *de novo* synthesis of cholesterol and its exogenous or dietary intake in the body (Gliozzi et al., 2021).

### Cholesterol

Cholesterol is a lipid molecule that belongs to the family of polycyclic compounds known as sterols. The term cholesterol is coined from Ancient Greek -*chole* (bile) and *stereos* (solid), suffixed by –*ol* for its alcohol group (Morzycki, 2014). Cholesterol accounts for 30–40% of the animal cell membrane composition, which is more than any other biomolecule (Pinkwart et al., 2019). Ever since its discovery and isolation from bile and gallstones by Poulletier de la Salle in 1769, as documented by Dam et al. (1959), cholesterol has fascinated physiologists, biochemists, and clinicians due to its physiological and pathological influence on normal cellular functions. Despite being structurally as well as functionally significant, the toxic accumulation of cholesterol in the cell in animals leads to debilitating effects on the whole organism. Cholesterol homeostasis is influenced not only by various intrinsic factors such as genetics, body weight, circadian rhythms and endocrine factors but also by external therapeutic and nutritional factors (Pallottini et al., 2004; Santosa et al., 2007; Alphonse and Jones, 2016).

### Cholesterol Biosynthesis

Although almost all animal cells synthesize cholesterol, the liver is the principal source of cholesterol synthesis and contributes to more than 50% of the body’s requirement (Repa and Mangelsdorf, 2000). Owing to the multi-faceted nature of cholesterol and the complexity of *de novo* synthesis, the latter has several checkpoints throughout its progression and the intermediates serve as precursors for bile, vitamin, and steroid hormones that play vital roles in other cellular processes (Cerqueira et al., 2016). Cholesterol biosynthesis is a concerted process that occurs in the ER involving more than 20 enzymes that are synthesized in the ER. An elaborate description of all the enzymes and steps involved in the pathway is not discussed in this review and the reader is referred to other reviews (Trzaskos and Gaylor, 1985; Howe et al., 2017). It can be subdivided into five major steps consisting of the mevalonate pathway, followed by a series of reactions that lead to the formation of isoprenes, squalenes and sterols. HMG-CoA reductase (HMG-CoR) involved in the last step of the mevalonate synthesis plays dual roles of being the rate limiting enzyme as well as the check point for feedback mechanisms. It is the most regulated enzyme in the body that is monitored and modulated at all stages, ranging from transcription and translation level to its activity and degradation. Accordingly, HMG-CoR is of immense therapeutic significance in a number of dyslipidemic disorders (Jiang et al., 2018).

Mevalonate is subsequently converted to isoprenes such as isopentanyl 5-pyrophosphate and dimethylallyl pyrophosphate. Further condensation results in the formation of a 30-carbon molecule, squalene, which serves as the precursor for all steroids. Squalene monooxygenase (SM), also known as Squalene epoxidase, catalyses the first oxygenation step of the process. It is the second rate-limiting enzyme of sterol synthesis pathway and has now started to gain attention as a key regulator of cholesterol synthesis (Gill et al., 2011; Yoshioka et al., 2020). The consequential modification of squalene produces the four –ring compound lanosterol that further transforms into cholesterol either by adopting the Bloch pathway or the Kandutsch-Russel Pathway through a series of complex mechanisms. While the former pathway uses Δ ^24^ unsaturated sterols and produces desmasterol which may be converted to cholesterol, the intermediate metabolites produced by the latter pathway are saturated sterols that eventually terminate in 7-hydroxycholesterol (Cerqueira et al., 2016). Thus, the fate of lanosterol is determined by the cell type in which it is synthesized and the cells’ requirement of steroids.

### Lipoproteins as Mediators of Cholesterol and Lipid Recycling and Transport

Due to its hydrophobic and cytotoxic nature, cholesterol is esterified at its hydroxyl group by ACAT 1 located in the ER and translocated to the plasma membrane and other peripheral tissues (Lange et al., 2014; Litvinov et al., 2018). While newly synthesized cholesterol transport occurs against a steep concentration gradient, with the expenditure of energy, retrograde transport of cholesterol from the plasma membrane and other peripheral tissues to the ER of hepatocytes for its esterification is non-energy dependent. The cholesteryl esters are either stored along with triglycerides as cytosolic lipid droplets in hepatocytes as well as other cell types or transported as lipoproteins with the aid of carrier proteins termed apolipoproteins (Brown and Goldstein, 1986). Lipid homeostasis in the tissue and plasma are regulated by these lipoproteins along with lipoprotein receptors, lipolytic enzymes and transfer proteins (Shepherd, 1994). At different stages of its life cycle, lipoproteins are referred to as chylomicrons, chylomicron remnants, low-density lipoprotein (LDL), very low-density lipoprotein (VLDL), intermediate density lipoprotein (IDL) and high-density lipoprotein (HDL) and Lipoprotein (a) [Lp(a)], based on their size, lipid concentration and apolipoproteins associated with it (Feingold et al., 2000).

In the liver, cholesteryl esters, triglycerides and phospholipids are packaged with the apolipoprotein B-100 (ApoB100) and released into the circulation as VLDLs (Ference et al., 2020). The lipid core comprised of sequestered cholesteryl esters and triglycerides is covered by a monolayer of phosphatidylcholine- the structural lipoprotein -lipoprotein. This packaging enables the transport and uptake of its contents with the aid of tissue specific cell receptors. The extrahepatic tissues recognize specific apolipoproteins on the lipoproteins and deplete the triglyceride levels in these lipoprotein complexes with the help of specific lipases. For instance, while lipoprotein lipase (LPL) acts on ApoB containing lipoproteins, hepatic lipases prefer Apo-A1 containing lipoproteins. Conversely, as the VLDLs progress to form LDLs in the plasma, it undergoes further modification characterized by a reduction in lipid core mass coupled with – the addition of ApoC2 and ApoE -thereby increasing its density. ApoE can also associate with HDL particles as the latter in size and decrease in density. Hence, VLDLs that target the adipocytes contain the highest lipid to protein ratio, compared to the IDL and LDL with intermediate and lowest levels, respectively (David, 2017).

Low-density lipoproteins are captured by the transmembrane LDL receptor (LDLR) that recognize the ApoB100 lipoprotein. LDLR is produced in the ER and matures in the Golgi complex (Goldstein and Brown, 2009). The LDL-LDLR complex is internalized in clathrin-coated vesicles to form early and late endosomes that eventually fuse with lysosomes to form endolysosomes. The acidic pH in the endosome favors the dissociation of LDLR from the complex and its recycling to the plasma membrane to continue the trafficking. The cholesterol esters are hydrolyzed to free cholesterol and fatty acids in the early endosomes and carried to the Golgi complex. The membrane bound- NPC1 and luminal NPC2 (Niemann-Pick C) proteins located in lysosomes regulate the Golgi to ER trafficking of cholesterol (Wang et al., 2010). Like HMG-CoR and SCAP (SREBP cleavage activating protein), these proteins also carry the sterol sensing domain through which they modulate cholesterol uptake. In addition to the NPC proteins, oxysterol binding protein (OSP)-related protein (ORP) family located on lysosomes also contribute to lysosome mediated sterol metabolism and transport (Soccio and Breslow, 2004; Wilhelm et al., 2017). Hepatocytes, macrophages and other peripheral cells that express the transmembrane LDLR are recipients of LDL cargo.

Dietary triglycerides and cholesterol are absorbed by the enterocytes that package the fats along with ApoC2 and ApoB48 apolipoproteins to form chylomicrons. The chylomicrons enter the plasma through the lymphatic system and are transferred to extrahepatic tissues that recognize the ApoC2 residues. After secretion, they acquire ApoE from HDL. The lipase-hydrolyzed, lipid and apolipoprotein-depleted chylomicrons containing ApoE are now called chylomicron remnants, that are released into the blood. These remnants, identified by their ApoE tags, are uptaken by the liver through the hepatic LDLR.

**FIGURE 1:**
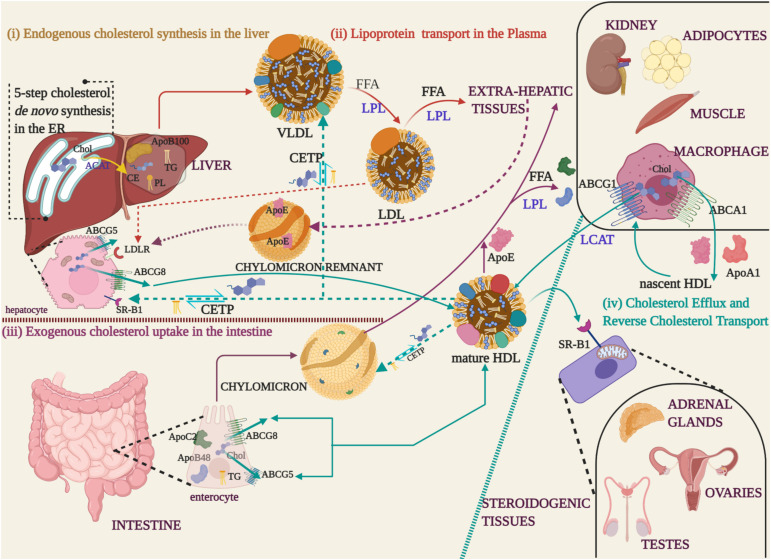
Cholesterol synthesis and lipoprotein transport **(i)** Endogenous cholesterol synthesized in the Endoplasmic Reticulum (ER) of liver is esterified to cholesteryl ester (CE) by Acyl-coenzyme A: cholesterol acyl transferase 1 (ACAT) (yellow arrow), packaged with phospholipids (PL), triglycerides (TG), and apolipoprotein B 100 (ApoB100) into Very Low-Density Lipoproteins (VLDL) for transport into the blood stream. **(ii)** During the course of lipoprotein transport in the plasma (red arrows), the free fatty acids (FFA) are hydrolyzed by Lipoprotein Lipase (LPL) and the apolipoprotein/CE depleted Low-Density Lipoproteins (LDL), recognized by their ApoB100, are uptaken by extra-hepatic tissues. **(iii)** Dietary fats absorbed by the enterocytes are packaged with ApoB48 and ApoC2 apolipoproteins along with TG to form chylomicron droplets that are released into the blood stream through the lymphatic system (Purple arrows). Extra-hepatic tissues recognize the apoproteins and take up the cholesterol by LPL-mediated hydrolysis of FFA. The cholesterol depleted chylomicron remnants carrying the ApoE apolipoprotein, acquired from HDL, are returned to the hepatocytes via the Low-Density Lipoprotein Receptor (LDLR) of hepatocytes (purple dotted arrows). (iv) Excess cholesterol from extra-hepatic tissues is released through ABC transporter proteins (ABCG5/ABCG8 -enterocytes and hepatocytes, ABCA1/ABCG1-macrophages) to High-Density Lipoprotein (HDL) that carry ApoE and ApoA1 apolipoproteins (cyan arrows). In macrophages, cholesterol effluxed through the ABCA1 transporter is packaged into nascent HDL. Nascent HDL triggers cholesterol esterification by LCAT and recruits more cholesterol through the ABCG8 transporter to form mature HDL. Mature HDL transfers cholesterol to steroidogenic tissues, through the scavenger receptor (SR-B1) located on the mitochondria, for the synthesis of steroid hormones and bile acids. The excess cholesterol is reverse transported from extrahepatic tissues and returned to the liver through the chylomicrons and lipoproteins (cyan dotted arrows). During the process, TG are exchanged for CE through the cholesteryl ester transfer proteins (CETP).

Cholesterol and triglycerides are brought back to the liver from peripheral tissues in HDL, the abundant lipoprotein in the circulation that contain the ApoA-I as its major protein component (Zannis et al., 2004; Hadjiphilippou and Ray, 2018). The protective property of HDL, commonly referred to as ‘good cholesterol’, is credited to its lipoprotein and lipid content. Excess cholesterol is disposed of most extrahepatic tissues as they lack the machinery to catabolize it. Cholesterol efflux from these sites is mediated by ATP-binding cassette (ABC) transporter proteins that are specific to certain cell types. ABC transporters are cell surface markers, of which ABCA1 (family A, member 1) and ABCG1 (family G, member 1) are located on macrophages. Hepatocytes and enterocytes harbor ABCG5 and ABCG8 (family G, member 5; family G, member 8) (Wang et al., 2015). The excreted cholesterol is acquired by the ApoA-1 to form nascent HDL. The maturation of HDL is marked by the esterification of free cholesterol by Lecithin: cholesterol acyl transferase (LCAT). Cholesterol esterification coupled with HDL enlargement qualifies mature HDL to recruit more cholesterol through ABCG1 transporter (Gelissen et al., 2006; Zimetti et al., 2015). In due course, the polar (free cholesterol and phospholipids) and non-polar lipids (cholesteryl esters, triglycerides) collected from peripheral various tissues, are distributed to steroidogenic tissues for steroid hormone and bile acid synthesis. Triglyceride-rich lipoproteins (TRGL) in the plasma function as energy banks by delivering free fatty acids to peripheral tissues (Walther and Farese, 2012). TRGLs include chylomicrons in the intestine, VLDL and IDL in the blood and their mode of operation of lipolysis is coordinated by LPL. HDL contributes to this energy supply through the reverse cholesterol transport (RCT). The exchange of cholesteryl esters for triglycerides between TRGLs and HDL is through the cholesteryl ester transfer protein (CETP). The HDLs return these lipid components to the liver where it is excreted as bile acids. In Figure 1, a schematic representation of lipoprotein mediated cholesterol trafficking is described.

## Lipid and Sterol Homeostasis

The constellation of events regulating cholesterol metabolism involve the alliance between various tissues and are not limited to the hepatocytes that contribute to the bulk of *de novo* cholesterol or the NPC1L1 (Niemann-Pick C1- Like1) expressing enterocytes of the intestine, where dietary cholesterol is absorbed. The ER houses most of the enzymes involved in cholesterol synthesis and regulates complex feedback loops, storage, trafficking and transport. Expectedly, an uneven distribution of cellular cholesterol is maintained, wherein 60–90% reside on the plasma membrane while ER exhibits less than 1% on its membranes (Luo et al., 2017). The distribution of sterols in the mitochondria resembles that of the ER, while the endocytic compartments, the *trans*-Golgi network and the Golgi apparatus falls between these extremes (Van Meer et al., 2008). The plethora of functions orchestrated by cholesterol in the body are attributed to its asymmetric compartmentalization that dictate the exchange of cholesterol between various tissues (Van Meer et al., 2008).

### Transcription Factor Mediated Regulation of Sterol Metabolism

By virtue of the complex nature of mechanisms involved in maintaining cholesterol balance in the body, its synthesis, storage, trafficking, uptake, and export are processes that are both strictly monitored as well as energy demanding (Ikonen, 2008). Mammalian cells regulate cholesterol levels in the body either by controlling (i) *de novo* cholesterol synthesis or (ii) LDLR mediated uptake of exogenous cholesterol sequestered in LDL from the blood (Brown and Goldstein, 1997; Goedeke and Fernández-Hernando, 2012). As elaborately reviewed by (Luo et al., 2020; Soccio and Breslow, 2004), cholesterol synthesis is tightly controlled and the key regulators include SREBP and two rate-limiting enzymes, HMG-CoR and SM. Members of the SREBP (Sterol Receptor Binding Protein) family play pivotal roles in cholesterol metabolism, of which SREBP2 and SREBP1a are the key modulators. While SREBP2 regulates cholesterol biosynthesis, SREBP1a and SREBP1c modulate fatty acid synthesis (Amemiya-Kudo et al., 2002; Horton et al., 2002). The ER membrane-embedded protein INSIG (Insulin induced genes) regulates the transcriptional and post translational activation of SREBP and HMG-CoR, respectively (Rawson, 2003; Burg and Espenshade, 2011). INSIG is either directly bound to the sterol sensing domains (SSD) of HMG-CoR or indirectly, via SCAP, to the post translational precursors of SREBP that are retained in the ER membrane. This interaction is sterol-dependent, wherein cholesterol binds to SREBP-SCAP complex and oxysterol binds to INSIG. Lanosterol and oxysterol regulate the activation of HMG-CoR mediated mechanisms through the E3 ubiquitin ligase (Zelcer et al., 2014). In sterol depleted cells, the SREBP-SCAP complex is released from INSIG-1, and the latter is degraded by the proteasome, until cholesterol levels are restored. The release of INSIG changes the conformation of the SCAP-SREBP complex and SREBP is escorted from the ER, via the COPII coated vesicles, to the Golgi apparatus, where it is cleaved and activated. The cleaved nuclear SREBP fragment homodimerizes and binds to the Sterol Response Elements (SRE) in the promoter regions of cholesterogenic genes and other targets that increase cholesterol uptake and biosynthesis in the body. Cholesterol metabolism is also regulated post translationally by inhibiting HMG-CoR. When cholesterol or oxysterol are in surplus, its synthesis is down regulated by the sterol-mediated, protein–protein interaction between SREBP-SCAP complex and INSIG-1, respectively. As a result, the activation of downstream targets such as *LDLR* and *HMG-CoR* genes are turned off. When lanosterol and oxysterol are in abundance, the INSIGs remain bound to HMG-CoR. This complex down regulates HMG-CoR by triggering the E3 ligase and proteasomal degradation mediated by the ERAD (ER associated degradation) machinery. In addition, oxysterols regulate the levels of the oxysterol receptor- LXR (Liver X Receptor) (Peet et al., 1998; Zelcer and Tontonoz, 2006; Guillemot-Legris et al., 2016).

### ER and Mitochondria Mediated Surveillance of Lipid Metabolism: The Involvement of UPR, ER Stress and Oxidative Stress

The dynamic roles played by ER ranges from Ca^2+^ signaling, protein folding, lipid and carbohydrate metabolism. As discussed in the section ‘Lipoproteins as mediators of cholesterol and lipid recycling and transport’ LDLR mediated internalization of LDL and cholesterol delivery to target tissues is - detrimental in maintaining stabilized lipid levels in the plasma as well as tissues. The synthesis of LDLR occurs in the ER and the internalization of LDL in the cells is proportional to the LDLR turnover. Being the major hub where nascent polypeptides are synthesized, the ER has its own quality control machinery (ERQC) comprised of ER chaperones, that ensure proper folding of the protein released from the ER, through the *cis-trans* Golgi network, to their site of action. Misfolded proteins are redirected into the chaperone complex until it is folded properly. Improperly folded proteins are eliminated by means of the ERAD pathway that directs it to the proteasomal degradation system. In line with the accumulation of misfolded proteins in the ER, the Unfolded Protein Response (UPR) halts further transcription and translation of the proteins. It is the signaling mechanism that promotes cell survival and is achieved by (i) halting further translation (ii) recruiting ERAD components to clear the unfolded protein (iii) upregulation of molecular chaperones that rectify the unfolded protein. Persistent activation of UPR propels ER stress in the cells that ultimately leads to apoptosis. The three major ER stress sensor proteins of UPR are (i) Inositol Requiring Enzyme 1 Alpha (IRE1A), Activating Transcription Factor 6 (ATF6) and (iii) Protein Kinase RNA-like ER kinase (PERK). In the normal state, the sensor proteins are maintained in the inactive state by binding to the ER chaperone, Glucose Response Protein-78 (GRP-78/BiP). Cellular stress, induced by internal and external cues, releases BiP from the ER sensors and activates these membrane-bound proteins to turn on the cascade of ER stress response. The release of BiP triggers the phosphorylation and dimerization of IREIA and PERK arms that impel specific transducers to execute subsequent functions. The active PERK facilitates the phosphorylation of eukaryotic translation Initiation Factor 2A (eIF2A) to inhibit general protein translation, but with the exception of selective mRNAs such as activating transcription factor 4 (ATF4). The attenuation of protein translation together with activation of ATF4 reduces the burden of post translational processing and trafficking of proteins, which in turn promotes the restoration of cellular homeostasis by upregulating genes involved in cell recovery However, prolonged stress induces ATF4- mediated expression of ER chaperones and C/EBP homologous protein (CHOP) (Hetz, 2012; Hu et al., 2019). Traditionally referred to as the translational arm, the PERK/eIF2A/CHOP arm regulate lipogenesis by controlling the processing and maturation of SREBP-1 and 2, C/EBPα, C/EBPβ and PPAR γ (Peroxisome proliferator activated receptor-gamma) (Lakshmanan et al., 2013). Increased levels of ATF4 and CHOP triggers the transcription of ATG genes (AuTophagy-related Genes), a pro-survival mechanism of the cell, to clear off misfolded proteins with the help of lysosomes (B’Chir et al., 2013; Zahid et al., 2020). However, chronic levels of ATF4 and CHOP due to persistent ER stress can induce cell death mechanisms by the activation of GADD34, ERO1 and Caspases (Rozpedek et al., 2016). CHOP-induced cell death is attributable to the suppression of the cell cycle regulator protein 21(p21/WAF1). Under normal conditions, p21 there exists a crosstalk between CHOP and p21 – an inhibitor protein that arrests the progression of cell cycle at G1 phase in a p53-dependent manner. This association is involved in the transition from adaptive UPR to proapoptotic pathway (Mihailidou et al., 2010).

The endoribonuclease activity of IRE1A mediates the splicing of a 26-nucleotide sequence of the X-box Binding Protein-1 (XBP1) mRNA, a basic leucine zipper transcription factor and translocation to the nucleus. Spliced XBP-1 (XBP-1s) mRNA promotes the activation of ERAD components, ER chaperones, lipogenic genes such as *SREBP-1c* and genes involved in phospholipid biosynthesis (Sriburi et al., 2007; Fu et al., 2011). Apart from splicing of XBP1, the RNase domain of IRE-1A is also involved in lipid anabolism by inducing Regulated IRE-1 dependent mRNA decay (RIDD). The spliced XBP1s protein upregulates a cascade of UPR-associated transcriptional events that promote protein folding and ERAD.

Unlike the PERK and IRE-1A arms of the UPR pathway, detachment of BiP leads to the translocation of ATF6 to the Golgi complex, where it is cleaved and activated by Site-1 Proteases (S1P) and Site-2 Proteases (S2P). The activated ATF6 fragment enters the nucleus and upregulates the transcription of ERAD components, along with the inhibition of SREBP2 mediated sterol synthesis (Horton et al., 2002; Volmer and Ron, 2015). To add on, the overexpression of nuclear ATF6 in proximal tubular cells of the kidney down regulates PPAR α (Peroxisome Proliferator Activated Receptor -Alpha), the key player involved in beta oxidation of fatty acids, causing lipotoxicity due to abnormal lipid droplet formation and accumulation (Jao et al., 2019). When cholesterol levels are elevated, a feedback loop is operational through the post-transcriptional regulation of its sterol-activated ERAD (DeBose-Boyd, 2018). The ER provides a platform for several signaling pathways and metabolically regulating events (Verkhratsky, 2005). Lipid metabolism plays a major role in maintaining the ER membrane function, disturbing the balance of which leads to ER stress and metabolic dysfunction.

Apart from the ERAD machinery, autophagy is an alternative cellular pathway for the degradation of misfolded or immature protein cargo that takes place in the major recycling cellular compartment – lysosomes. There are three major types of autophagy namely macro-, micro- and chaperone mediated autophagy (CMA) (Parzych and Klionsky, 2014). In macroautophagy, the cargo is engulfed by double-membraned vesicles called autophagosome (AP) which eventually fuses with the lysosome, under the influence of ATG genes (Ohsumi, 2014). Macroautophagy can be categorized into non-selective or selective, depending on the target assigned for degradation. While non-selective macroautophagy targets the general cytosol, or selective macroautophagy degrades specific organelles - mitochondria (mitophagy), ER (ER-phagy), and peroxisomes (peroxiphagy) (Lipatova and Segev, 2015). Prolonged ER stress triggers macroautophagy where it functions as a backup for ERAD (Houck et al., 2014). Compromised ERAD or proteasome functions trigger autophagy through UPR-dependent components providing substantial evidence for the tight physiological relationship between these machineries (Fujita et al., 2007). For instance, overexpression of EDEM1 – a component of the ERAD, is capable of bypassing the proteasomal degradation pathway by forming amyloid-like oligomers that recruit the macroautophagic machinery for degradation (Chiritoiu et al., 2020). In microautophagy, ingression of cargo into lysosomes is marked by the invagination of the lysosomal membrane. CMA is a highly specific phenomenon restricted to mammalian cells and involves the translocation of unfolded proteins through the lysosomal membrane (Kaushik and Cuervo, 2019). Microautophagy and CMA do not depend on ATGs and AP for the delivery of their cargo. When the severity of stress exceeds the degradation capacities of ERAD and autophagy, the cell switches to apoptotic cell death. The ubiquitin-proteasome/ERAD-I or the autophagy-lysosome/ERADII system are two major cellular clearance machineries that govern protein quality in eukaryotic cells. Poor protein quality control mechanisms derail the metabolic homeostasis substantiating the pivotal role of proteotoxicity in disease pathology. Stearing therapeutics toward autophagy, with the aid of pharmacological or natural agents, furnish novel schemes for the management of metabolic diseases (Zhang et al., 2018).

In addition to the ER, the mitochondria play a vital role in contributing to the lipid homeostatic network (Tamura et al., 2020). Cholesterol is converted to steroids, bile acids and oxysterols in the mitochondria of steroidogenic tissues (Russell, 2003; Martin et al., 2016). It is implicated that the mitochondria take up HDL cholesteryl esters through the scavenger receptor B1 (SR-B1) for adrenocorticoid steroidogenesis (Fu et al., 2011; Hoekstra, 2017). Located on the mitochondrial inner membrane are two major cholesterol metabolizing enzymes the P450 side chain cleavage system (P450scc/Cyp11A1) and sterol 27-hydroxylase (Cyp27) (Okuda, 1994; Miller and Strauss, 1999). While P450scc is specific to steroid hormone producing cells, Cyp27 is widely expressed in all cells. It mediates the conversion of cholesterol to 27-hydroxycholesterol, the most abundant oxysterol in the plasma. Likewise, it performs several other functions as that of a repressor of SREBP processing, an LXR agonist and as a more soluble and easily transported form of cholesterol in the plasma (Russell, 2000; Fu et al., 2001). The ER and mitochondria are key integrators of signals arising from different pathways and regulate cellular stress and inflammation. The crosstalk between these two organelles involves sensing Ca^2+^ flow. While ROS in the mitochondria is generated as a byproduct of oxidative phosphorylation, disulfide bond formation during protein folding generates ROS in the ER. In fact, these two organelles together contribute to the majority of ROS in cells (Malhotra and Kaufman, 2011). Excess ROS leads to oxidative stress in the mitochondria and induces ER stress due to disturbances in redox biology in the ER lumen (Cao and Kaufman, 2014). ER stress and oxidative stress interact further via PERK/ATF4 signaling via calcium and ROS until the transcription factor for antioxidative response, NRF-2 is activated. Eventually, ER and oxidative stress leads to the activation of inflammatory signaling pathway that aggravates the pathological outcome manifested in various metabolic diseases (Chaudhari et al., 2014). While the NF-kB pathway are upregulated by all three branches of UPR, the IRE1A arm is responsible for the JNK cascade.

ER-mitochondrial tethering is profoundly studied and does not involve membrane fusion between these organelles. The interaction is mediated by enzymes of lipid synthesis, signaling molecules, and membrane proteins. The proteins localized to this sub-compartment are referred to as mitochondria-associated ER membrane (MAM) (Area-Gomez et al., 2012). The dynamics of the proteins located in this structure determine the ER-mitochondria function. The classic proteins include Mitofusin 1/2 (MFN1/2), ER chaperones (BiP, Calnexin and Calreticulin), 75 kDa glucose-regulated protein (GRP75), Oxysterol-binding protein (OSBP)-related proteins (ORP5 and ORP8) Voltage-Dependent Anion-selective Channel protein 1/2 (VDAC1/2), and Inositol 1,4,5-tris Phosphate Receptor type 3 (IP3R3) (Naon et al., 2016; Veeresh et al., 2019). Post-translational modification and interaction with other proteins, including MAM components, influence the ER-mitochondria contacts. Copious amount of PERK is found in the MAM (Verfaillie et al., 2012). Mitochondria-ER contacts coordinate lipid composition, whereupon MAM play a crucial role in sensing nutrient levels and maintaining Ca^2+^ homeostasis and ROS. Abnormal retention of proteins in the MAM lead to the disruption of calcium homeostasis, metabolic disorders and cell death (Liao et al., 2020; Yang et al., 2020).

Despite the accumulative knowledge on genetic factors that contribute to lipid homeostasis, the influence of epigenetic factors have also been reported by various groups (Ferrari et al., 2012; Meaney, 2014). It is notable that apart from the reports on the classical transcription factor-mediated regulation of lipid metabolism, a distinct category of regulators comprised of microRNAs (miRNAs), long non-coding RNAs (lncRNAs) and RNA binding proteins (RBPs) has emerged but is vaguely explored (Kapranov et al., 2007; Rottiers and Näär, 2012; Chen, 2016).

## Lipid Metabolism and Associated Diseases

A large volume of clinical, experimental and epidemiological data are available on lipid-associated disorders caused by external factors, lifestyle and inborn errors of metabolism. As per the lipid theory of diseases, LDL being the main transporter of serum cholesterol, is the primary determinant of abnormal accumulation of lipids in diseased conditions (Eikendal et al., 2016; Venugopal and Jialal, 2020). Likewise, biochemical modification of low-density lipoproteins are pathogenic hallmarks in various diseases (Alique et al., 2015). Lipid modifications alter the composition of LDL and can be categorized into (i) Oxidized LDL (oxLDL) (Schuh et al., 1978; Sawamura et al., 1997), (ii) glycated LDL (gLDL) (Vlassara, 2001), (iii) acetylated LDL (acLDL), (iv-v) ethylated and methylated LDL (Steinbrecher et al., 1984), and (vi) carbamylated LDL (cLDL) (Chen et al., 2020). These modifications alter the ApoB apolipoprotein and impair the LDL-LDLR interaction (Avogaro et al., 1991).

### Oxidized LDL

**FIGURE 2:**
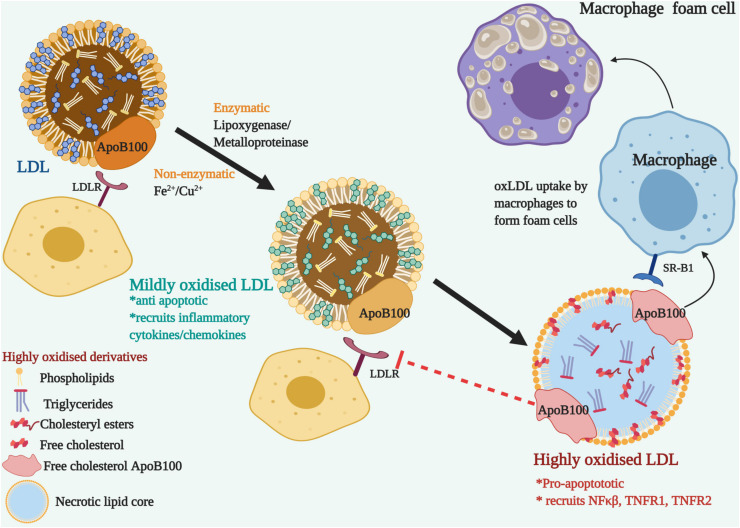
The transition of LDL to Oxidized Low-Density lipoproteins (ox-LDL)-Nascent LDL are recognized by LDL receptor-bearing cells through their ApoB100 apolipoprotein and do not get modified in the blood plasma due to the surveillance of the antioxidants. On the arterial walls, enzymatic or non-enzymatic modification oxidizes the lipoprotein components. Mildly oxidized LDL, though modified, can still bind to LDLR and recruits inflammatory cytokines and chemokines. LDLR fails to recognize the modified ApoB100, but it is recognized by the scavenger receptor, SR-B1 located on macrophages. Ox-LDL accumulated macrophages develop into foam cells. Pro-apoptotic and inflammatory signals are triggered with the recruitment of NFκβ and TNFRs.

Noteworthily, of all the lipid modifications discovered, oxLDL has been identified as the primary risk factor for cardiovascular diseases (CVD) and dyslipidemia (Itabe, 1998; Ke et al., 2018). Combination with other modifications such as gLDL, oxLDL contributes to the double modification phenomenon and expedites pathophysiological complications by the oxidation of existing glycosylated LDL (Rabini et al., 1994; Wu et al., 2008). Since the LDL itself is armed with an antioxidant component, alpha tocopherol or vitamin E, this along with the innate antioxidant defense mechanism rules out the occurrence of oxLDL in the circulation (Stocker and Keaney, 2004; Steinberg, 2009). However, lipid oxidation takes place either non-enzymatically, by transition metal ions (iron and copper) or through several enzymes (lipoxygenase and metalloproteinase) on the arterial walls. It occurs in two stages and produces mildly oxidized or highly oxidized LDL, depending on the degree of oxidation (Berliner et al., 1995; Steinberg, 2009). The first stage is marked by very little or no changes to LDL ApoB100 and the LDLR recognition and binding properties are not affected. Albeit, it brings about inflammatory changes by recruiting chemokines and cytokines, together with the activation of anti-apoptotic pathways and reducing the negative charge on the mildly oxidized LDL. The recruitment of inflammatory cytokines and chemokines causes further oxidation of LDL and protein modification of ApoB, as it transitions to the highly oxidized stage (Rhoads and Major, 2018). Apoprotein moieties of LDL are oxidized by oxidants at the basic, aromatic or sulfur-containing side chains (Ismael et al., 2015; Afonso and Spickett, 2019). Highly oxidized LDL are pro-apoptotic and fail to be recognized by LDLR (Steinberg, 2002). Instead, it attracts the scavenger receptors on macrophages that uptake oxLDL and form macrophage foam cells (Goldstein et al., 1979; Maiolino et al., 2013). Figure 2 describes the transition of LDL to highly oxidized LDL. Highly oxidized LDL are the pathogenic signature molecules found in macrophage foam cells and atherogenic lesions (Kluger, 1997; Steinberg, 2009; Di Pietro et al., 2016). Macrophages, smooth muscles and endothelial cells can generate oxLDLs. Interestingly, it was observed that the incubation of macrophages with oxLDL but not with native LDL leads to cholesterol ester accumulation (Parthasarathy et al., 2010; Hutchins et al., 2011). In addition, oxLDL-mediated apoptosis is characterized by a cascade of signaling events involving regulatory Bcl-2 gene family, caspases, sphingomyelinases and a wide range of nuclear transcription factors like NF-KB, TNFR1, and TNFR2. The core components of LDL -cholesteryl esters, triglycerides, phosphatidylcholine, undergo lipid peroxidation to form hydroperoxide and aldehyde derivatives (Girotti, 1998; Orsó et al., 2011). The content of unsaturated fatty acids such as linoleate and arachidonate determine the degree of peroxidation (Niki, 1997). The toxic oxysterols and lipid derivatives elicit reactive oxygen species (ROS) generation and alter cellular protein and lipids that trigger the formation of a necrotic lipid core.

### Other LDL Modifications

gLDL modifications occur due to the non-enzymatic addition of glucose or its metabolites to the positively charged lysine residues located on the LDL-receptor binding domain of LDL ApoB. As a result, LDL loses its electropositivity and fails to be recognized by the LDLR, causing prolonged retention of gLDL in the plasma (Soran and Durrington, 2011). Elevated levels of Advanced Glycosylation End-products (AGE) on LDL are manifested in diabetic conditions than in normal individuals and is governed by glucose concentration as well as duration of exposure (Goh and Cooper, 2008). Furthermore, glycated LDL is prone to oxidation and thus gLDL or highly oxidized gLDL (HOGL) increases the susceptibility to atherogenesis (Siddiqui et al., 2019). The spontaneous carbamylation of amine residues on LDL ApoB by urea- or thiocyanate-derived cyanate generates cLDL (Kraus and Kraus, 2001). Urea, escalated in chronic renal failure and thiocyanate, released in the blood in tobacco smokers, cLDL that increases the atherogenic risks in these subjects (Ok et al., 2005; Wang et al., 2007).

## Oxidized LDL and ER Stress in Human Diseases

Although the individual contributions of oxLDL and ER stress to the progression of a wide array of diseases have been elaborately studied and reviewed, reports on the collective involvement of these two factors in human diseases are limited. The following sections are aimed at identifying possible interactions between these two factors that may ultimately unveil common targets and biomarkers with therapeutic implications.

### Atherosclerosis and Other Cardiovascular Diseases

Cardiovascular diseases account for the highest morbidity and mortality rates worldwide. The identification of atherosclerosis as a major CVD dates to >3500 years ago, with the same clinical impression as seen in modern times (Shattock, 1909). It is characterized by chronic inflammatory responses that lead to the proliferation of smooth muscle cells and the formation of lipid-accumulated plaques along the arterial walls that leads to the narrowing of arteries (Bruemmer and Law, 2003). Several lines of authentication point to the identification of oxLDL as the risk factor and the biomarker for atherosclerotic CVDs (Liang et al., 2012). However, a standard clinical reference value of oxLDL in healthy patients is not available and varies in different studies (Lara-Guzmán et al., 2018). It is well-documented that the uptake of ox-LDL, produced by ROS-induced oxidative stress, through the scavenger receptors of macrophages leads to the formation of atherogenic plaques (Frostegård, 2013; He and Zuo, 2015). The rupture of these atherosclerotic plaques induces the formation of thrombus that leads to heart attack and stroke (Orekhov and Ivanova, 2016). Studies on the association of ER with atherosclerotic plaques and its rupture have shown that UPR is activated in early stages of atherosclerosis, which switches to PERK and CHOP mediated cell death mechanism in the later stages of atherosclerotic smooth muscles and macrophages (Malhotra and Kaufman, 2007; Zhou and Tabas, 2013). Studies based on human coronary autopsy samples revealed significantly high levels of GRP78 and CHOP expression (Myoishi et al., 2007). Recent studies report a strong association of Grp78/BiP with carotid plaques and implicates the clinical application of circulating Grp78/BiP as a marker of metabolic and cardiovascular risk (Girona et al., 2019). The incubation of HMEC-1 cells with mildly oxidized LDL activated the PERK, IRE1A and ATF6 arms of UPR and ER stress response as evident by the continuous expression of CHOP. siRNA mediated inhibition of the IRE1A arm, together with JNK pathway inhibitor, SP600125 revealed the involvement of IRE1A/CHOP and JNK/SAPK pathway in triggering oxLDL induced apoptosis (Schroeter et al., 2001; Napoli, 2003). The ER stress markers colocalized with 4-hydroxynonenal (4-HNE), lipid peroxidation marker, in atherosclerotic lesions and were upregulated in oxysterol and peroxide derivatives of oxLDL; namely 4-HNE and 7-ketocholesterol (7-Ketochol), also known to induce CHOP/JNK pathway mediated apoptosis (Pedruzzi et al., 2004; Myoishi et al., 2007; Sanson et al., 2009). Oxysterols impart detrimental perturbations to the endothelial cells by altering the lipid composition and ER protein folding thereby activating UPR (Luchetti et al., 2017). The expression and anti-apoptotic function of the ER chaperone ORP150 is widely reported in drug induced hypoxia, diabetes, neuronal ischemia and atherosclerosis (Kusaczuk and Cechowska-Pasko, 2013; Lindholm et al., 2017; Nam and Jeon, 2019). The ER chaperone ORP150 colocalizes with IRE1A in these lesions and were found to limit CHOP mediated apoptosis in the vascular walls via the phosphatidyl inositol 3-kinase/Akt signaling pathway.

Muller et al. (2011) have identified that oxLDL treatment of human vascular endothelial and smooth muscle cells triggers the intrinsic mitochondrial apoptotic pathway due to sustained increase in Ca^2+^ ions (Sanson et al., 2009). As a follow-up study on human endothelial cells, the same group has identified that oxLDL induced ER stress triggers autophagy as a pro-survival mechanism, independent of the apoptotic pathway activated by oxLDL. Sustained ER stress activates the pro-apoptotic arms of ER stress response through CHOP and JNK pathways (Ron and Walter, 2007). Interestingly, the report unveils the anti-apoptotic protective role of HDL in oxLDL-induced ER stressed cells and proposes that its mode of action is mediated by inhibiting the rise in calcium levels, a trigger for oxLDL and ER stress induced apoptosis and autophagy (Muller et al., 2011). Incubation of human endothelial HMEC-1 and U937 macrophagic cell lines with oxLDL inhibited the protein folding ER chaperone - Protein Disulfide Isomerase (PDI) and its modification. The inhibitory effect of oxLDL was mimicked by exogenous 4-HNE and prevented by *N*-acetyl cysteine (Muller et al., 2013; Nègre-Salvayre et al., 2017). *In vitro* studies on oxLDL – treated HUVEC cells demonstrated the activation of ER stress via JNK/CHOP pathway and mitochondria-mediated apoptosis (Tao et al., 2016). Zhou and his group reported the protective function of Nuclear Factor I A (NFIA) on oxLDL - induced ER stress and apoptosis in HUVEC cells (Zhou et al., 2019). The pro-apoptotical signal PKCδ was identified as a crucial player in the progression of inflammatory ER stress response involving CHOP and JNK induction. PKCδ regulates oxLDL induced ER stress through the IREA/JNK pathway (Larroque-Cardoso et al., 2013). Studies on a rat model by Liu et al. revealed that persistent ER stress induced Ca^2+^ overload leads to the activation of the Calcium-Sensing Receptor (CaSR) and activates Protein kinase C δ (PKC δ) mediated apoptosis (Liu and Green, 2019; Ajoolabady et al., 2021). Ample evidence from various research groups corroborate microRNAs as key modulators of oxLDL-mediated signaling in atherosclerosis (Zhang and Wu, 2014; Xu et al., 2015). The miR-33 family of miRNA regulates genes involved in HDL metabolism and cholesterol efflux, particularly ABCA1, which is downregulated by ER stress. An atherosclerotic model system of macrophages exposed to oxLDL established the pathological link between elevated miR-33 expression and ER stress-induced lipid metabolic disorders. Knock down of CHOP alleviates ER stress and mitigates lipid metabolic disorders in atherosclerotic conditions, hence signifies CHOP as a potential therapaeutic target (Sun et al., 2018; Zahid et al., 2020) the UPR and ER stress response arms for therapy has burgeoned in the recent past, however, successful clinical trials are lacking as many of these targets do not directly target any upstream ER regulatory checkpoints (Subach et al., 2009; Ren et al., 2021).

### Diseases of the Liver-NASH, NAFLD

The incidence of metabolic liver diseases like alcoholic or- non-alcoholic fatty liver disease (NAFLD) and its progressive component non-alcoholic steatohepatitis (NASH) is on the rise with an alarming increase in demand for liver transplantations (Younossi et al., 2018). NAFLD is clinically diagnosed by the abnormal accumulation of fat in the liver, with age > 50 years, T2DM and obesity being its risk factors (Anstee et al., 2013). The progression of NAFLD to NASH is attributed to ER stress and mitochondrial dysfunction (Hotamisligil, 2010; Ozcan and Tabas, 2012; Henkel and Green, 2013; Maiers and Malhi, 2019). Together with the risk factors associated with NAFLD and NASH, it increases the susceptibility toward developing cirrhosis, hepatocellular carcinoma (HCC), CVD or even chronic kidney diseases (CKD) (Galle et al., 2018; Sun et al., 2018). Taking into account the range of organs or tissues affected, NAFLD or hepatic steatosis can be called a multisystem disease (Byrne and Targher, 2015; Wong et al., 2016). oxLDL leads to liver injury and is used as a serum biomarker in patients with non-alcoholic steatohepatitis, hepatitis C infection, alcoholic and non-alcoholic liver diseases (Schroder et al., 2006; Nakhjavani et al., 2011; Ampuero et al., 2016). A growing body of information suggests that oxLDL contributes to hepatic inflammation and the progression of chronic liver diseases by the abnormal accumulation of oxLDL in the Kupfer cells, also called hepatic macrophages (Bieghs et al., 2013; Ho et al., 2019). Alcohol induced ER stress hepatic stress has been established along the PERK/FOXO3 and ATF6 arms as a mechanism for the pathogenesis of liver diseases in rodents and humans (Ji, 2014). While a vast majority of studies target the canonical ER stress response sensors that attenuate protein translation, or the downstream apoptotic pathway, Shin et al. (2013) identified the translational repressor function of SIRT7 as a potential therapeutic ER stress alleviator and hepatic homeostatic regulator. SIRT7, a histone H3 lysine 18 (H3K18) deacetylase belonging to the sirtuin family, is recruited by the XBP-1 arm of UPR to block gene expression and reduce ER stress. The XBP-1 recognizes specific nucleotide sequences on the promoter region of SIRT7 and the latter targets genes involved in protein translation and ribosome biogenesis. The SIRT7 interaction on the promoter region of these target genes is stabilized by Myc, a master regulator of ribosome biogenesis. They used a murine SIRT7 knock-out model to demonstrate that aggravated NAFLD-induced ER stress can be reverted by overexpression of SIRT7 in high-fat diet fed mice (Shin et al., 2013). It is interesting to note that a similar study using another strain of mice reported that SIRT7 deficient mice were resistant to steatosis at 12 weeks of age and did not develop fatty liver as they matured (Yoshizawa et al., 2014). The ambiguity arises, as justified by the authors, due to the difference in mice strains and the experimental protocol. Hence it cautions that the variation in experimental design alters the interpretation of results and is a caveat in identifying potential therapeutical targets.

Lectin-like oxidized low density lipoprotein-receptor (LOX-1) is a specific cell-surface receptor for oxLDL located on macrophages and liver (Zhang et al., 2015). Activation of IRE1/XBP-1 signaling pathway upregulates LOX-1expression and contributes to oxLDL-induced foam cell formation in macrophages (Ishiyama et al., 2011). An interesting study on the liver cell line L02 suggest that down regulation of LOX-1 by ER stress can be neutralized by rescuing the cells with low or medium doses of HDL, thereby improving lipid uptake in the liver cells. Competitive inhibition of oxLDL by upregulation of LOX-1 receptor in the liver helps to lower plasma oxLDL and thus reduces atherosclerotic risks (Hong et al., 2015). Although this study was conducted with the objective of reducing oxLDL-induced cardiovascular risks, the strategy can be adopted to combat liver diseases contributed by oxLDL and ER stress.

A major challenge that limits our understanding of the progression of metabolic diseases in human is that paramount data generated on oxLDL-induced ER stress associated disorders of the liver and therapeutic targets are based on animal models. Likewise, several research groups have successfully identified targets afflicted in liver diseases that can be salvaged by pharmacological interventions, the pathological link between oxLDL and ER stress is overlooked as they are confronted from different perspectives (Lebeaupin et al., 2018; Chadwick and Lajoie, 2019; Burchill et al., 2021).

### Type II Diabetes Mellitus (T2DM), and Diabetic Retinopathy

The onset of Type II Diabetes mellitus is marked by defective secretion of insulin and apoptotic loss of the islet cells (Richardson et al., 2013). Genetic predisposition together with systemic insulin resistance, chronic levels of cholesteryl esters and modified LDL trigger beta cell damage and increase the risk of CVDs (Cleland, 2012; Ludwig et al., 2014). Apart from the presence of highly oxidized LDL and anti-oxLDL antibodies in T2DM, modified oxLDL receptors in human islet cells have also been confirmed by various groups (Alouffi et al., 2018; Santiago-Fernández et al., 2019; Xie et al., 2019; Puchałowicz and Ra′c, 2020). Pancreatic cells are more prone to ROS-induced oxidative stress as these cells have limited intrinsic antioxidant defense mechanisms. ER and oxidative stress induce insulin resistance and T2DM, characterized by multi-organ dysfunction including the liver, adipose tissue, pancreas and brain. In the adipocytes, fatty acid and glucose trigger IRE1A activation - leading to insulin resistance through the JNK signaling cascade, and PERK arm, which triggers the secretion of adipokines, Tissue Necrosis Factor Alpha (TNFA) and Interleukin-6 (IL-6). These ER stress signals released from adipocytes exasperate ER stress in beta islet cells of the pancreas. The progression of T2DM in the pancreatic islets follows the PERK/eIF2A/ATF4/CHOP mediated apoptosis, coupled with reduced glucose-stimulated insulin secretion (Ghemrawi et al., 2018). Co-culture experiments using insulin secreting cells (MIN6) with human pancreatic islets from biotherapies and cadaver tissues led to the finding that oxidative stress induced oxLDL imparts beta cell dysfunction (Favre et al., 2011). Mildly oxLDL activated the IRE1A-JNK-CHOP expression mediated apoptosis in beta cells, in line with previous reports (Xu et al., 2005). Though the PERK arm can also mediate CHOP activation via ATF4, the upregulation of P58IPK, the inhibitor of PERK, substantiated the involvement of the IRE1A arm. In addition, CHOP and P58IPK expression was attenuated in *N*-acetyl cysteine (NAC) and 4-phenyl butyric acid (4-PBA) treated cells, supporting the hypothesis of therapeutic effects of antioxidants to combat T2DM and beta cell dysfunction (Hamer and Chida, 2007; Plaisance et al., 2016). An interesting recent finding by Wu et al. have elucidated down regulation of ox-LDL induced ER stress response activation, via PERK- peIF2A-ATF4-CHOP-Caspase 3 mediated cell death, by Secretagogin (SCGN), a Ca^2+^ sensor protein. Biochemical and bioinformatic tools identified the protein–protein interaction between ATF4 and SCGN. MIN6 cells recovered from oxLDL induced ER stress when treated in combination with SCGN and PBA. Intriguingly, the activation of CHOP by the IRE1A arm was not evaluated (Wu et al., 2021). oxLDL imparts a negative impact on insulin sensitivity and beta cell function in adults and elevated levels of circulating oxLDL are associated with preeclamptic pregnancy and fetal growth restrictions (Uzun et al., 2005; Holvoet et al., 2008; Maisonneuve et al., 2015). The low levels of expression antioxidative enzymes and high oxidative energy requirements of pancreatic-beta cells are held responsible for the threat posed by oxLDL on the islet cells (Shah et al., 2007). A cohort study on cord blood specimens identify a negative relation between oxLDL and beta cell function in fetuses and newborns (Fang et al., 2021).

High concentration of oxidized LDL imparts insulin resistance and adds to the development of MetS (Linna et al., 2015). On the contrary, a cohort study on non-diabetic subjects without clinical symptoms for atherosclerosis, mediation analysis was used to identify oxLDL-mediated correlation between obesity and insulin resistance with MetS. It affirms that insulin resistance and obesity occur, in parallel, as cardiometabolic risk factors for dyslipidemic conditions (Hurtado-Roca et al., 2017). Diabetic Retinopathy accounts for the major cause of blindness in diabetic patients worldwide. The pathogenesis is associated with neuronal and vasculature abnormalities due to death of retinal ganglion cells (Barber et al., 1998). Lipid peroxidation products are a major source of oxidative stress that induce hyperglycemia along with dyslipidemic conditions that cause the modification and leakage of serum lipoproteins through the blood retinal barrier (BRB) propagates Diabetic Retinopathy. Analogous to atherosclerosis, extravasation and oxidation of LDL were identified by immunohistochemical techniques in retinal Müller cells of retinal cells of diabetic patients, even in clinically normal retinal capillaries (Wu et al., 2008). HOGLDL induces ER stress in cultured human retinal capillary pericytes (HRCP), by upregulating all three arms of UPR, as evident by the presence of p-eIF2A, truncated ATF6, increased XBP1s mRNA and the proapoptotic mediator, CHOP. Likewise, ER stress markers ATF6 and GRP78 were detected in the human diabetic retina (Fu et al., 2012). oxLDL-Immune Complexes (oxLDL-IC) are more potent inducers of ER stress than oxLDL in human retinal pericytes and triggers CHOP mediated apoptosis in diabetic retinopathy by means of the CD36 receptors (Fu et al., 2014).

Various knock-out rodent models have provided a deep insight on the onset and advancement of T2DM. The ensuing to gain- or-loss-of function of key factors that are spatiotemporally expressed vary in animal and human models due to differences in the differentiation processes. Over the past decade, a unique platform for disease modeling that couples genome editing with human iPSC-derived B cells is a promising approach because of its species-and disease-specificity.

### Obesity

Similar to T2DM, obesity is the disease of the 21st century. According to the World Health Organization (WHO), the prevalence of obesity has tripled in the past 40 years and is no longer considered to be a disease of the affluent. The onset of obesity occurs due to inflammation and dysfunction of the adipose tissue. The adipose tissue is a reservoir of energy and secretes adipokines (Visfatin and resistin) and chemokines TNFA and IL-6 (Hug and Lodish, 2005; Wang et al., 2009, 2020; Harvey et al., 2011). Escalated ER stress in the adipose tissue of obese individuals modifies the adipokines and increases inflammation (Boden and Merali, 2011; Cao and Kaufman, 2014). Hypercholesterolemia leads to obesity by the accumulation of ox-LDL in the adipocytes that triggers ER stress mediated inflammation. The excessive secretion of adipokines induce insulin resistance, endothelial dysfunction to exuberate cardiovascular complications and T2DM (Guh et al., 2009). Molecular factors associated with fat cell defects include mitochondrial dysfunction, increased autophagy and the induction of oxidative and ER stress (Blüher, 2013). Studies in 3T3-L1 adipocyte cell lines have demonstrated that the oxLDL activated CHOP pathway was inhibited by tauroursodeoxycholic acid (TUDCA) (Chen et al., 2013). Proteosomal inhibition by MG-132 treatment of 3T3-L1 cells along with oxidative damage and ER stress contribute to insulin resistance in obesity (Díaz-Ruiz et al., 2015). oxLDL significantly increased the expression of the inflammatory chemokine Monocyte chemoattractant Protein-1 (MCP-1) in 3T3-L1 adipocytes. The Apo-A1 mimetic peptide L4F inhibited oxLDL mediated secretion in a dose dependent manner and attenuated MCP-1 expression through the C/EBPβ signaling pathway (Xie et al., 2016). Song et al. (2016) used an *in vitro* ox-LDL induced adipocyte model to demonstrate that HDL blocked ox-LDL-induced ER stress-CHOP pathway-mediated adipocyte inflammation by upregulating the expression of the scavenger receptor SR-B1 on adipocytes. They identified that the activation of CHOP was directed by the PERK and ATF6 arms of UPR (Song et al., 2016).

For the reason that intricate crosstalks exist between the underlying molecular mechanisms of T2DM, obesity and hepatic – related metabolic diseases, determining the linchpin of a specific pathway in this vicious cycle is a perplexing and cumbersome task.

### Non-metabolic Disorders- Alzheimer’s Disease and Cancer

In addition to the metabolic diseases discussed in the previous sections substantiating the interplay between oxLDL and ER stress, these two factors are also involved in the progression of non-metabolic diseases. There are a variety of protein misfolding disorders (PMD) that affect the nervous system. Alzheimer’s disease (AD), Amyotropic Lateral Sclerosis (ALS) and Parkinson’s disease are categorized under this class of diseases with a characteristic long clinically silent phase (Soto, 2003; Selkoe, 2004). Beta amyloid proteins are the neurotoxic hallmark protein of AD. The formation of beta amyloid plaques in the temporal lobe lesions is closely related to oxidative stress that modifies the cholesterol content of these pathogenic signature proteins (Gamba et al., 2012; Wang et al., 2014). Serum oxLDL has been implied as a biomarker for Alzheimer’s disease (Sun et al., 2003; Bacchetti et al., 2015). AD patients had significantly higher levels of oxLDL in their plasma compared to the control group and positively correlated with the severity of AD (Wahrle et al., 2002; Dildar et al., 2010; Ishizuka et al., 2020). The toxic accumulation of the beta amyloid proteins elicit the ER stress response, ensuing synaptic dysfunction and neurodegeneration thereafter (Duran-Aniotz et al., 2014). The coalescence of upregulated chaperones, phosphorylated PERK-eIF2A-CHOP proteins chaperones and the build-up of ubiquitinated proteins are the salient features of ER stressed condition in AD (Van Leeuwen et al., 1998; Stutzbach et al., 2014; Duran-Aniotz et al., 2017). Activation of PERK arm of UPR together with abnormal phosphorylation of tau in neurons are reported in AD (Hoozemans et al., 2009; Nijholt et al., 2012). Inhibition of PERK by the oral administration of a PERK inhibitor, GSK2606414 in a transgenic mouse model of Fronto Temporal Dementia (FTD) prevented tau-mediated neurodegeneration (Radford et al., 2015). IRE1A deletion in a transgenic mouse model helped to restore learning and memory capacity and reduced amyloid deposition (Duran-Aniotz et al., 2017). Recently, preclinical models of pharmacological intervention or gene therapy approaches that target the UPR have promising therapeutic applications for slowing down AD (Ohno, 2014; Scheper and Hoozemans, 2015; Cissé et al., 2017). However, very few documentations point toward the interplay between oxLDL and ER stress mediated neurodegenerative diseases and carcinogenesis. Hence, therapy based on animal or human models in this line is still in its infancy. oxLDL links neurodegenerative disorders and cancer, particularly glioblastoma and prostate, liver and colon cancer (Chen et al., 2015). Serum levels of oxLDL are elevated in breast and ovarian carcinoma and thus can be used as a measurable risk factor (Barclay et al., 1970; Gadomska et al., 1997). Targeting CD36- a oxLDL receptor, has therapeutic implications in preventing metastasis (Pascual et al., 2017). Administration of oxLDL augments proliferation in ovarian cancer cell lines and reduces the IC_50_ of cisplatin (Scoles et al., 2010). Hence, identifying therapeutic targets that curtail the effects of oxLDL can help in reducing chemoresistance to drugs. Similarly, as suggested by Lin et al. (2019), shifting the targets for therapy from the current strategies aimed at chemotherapy- based cell killing, to the cryoprotective UPR elements is a promising approach for tumor treatment.

## Conclusion and Future Perspectives

**FIGURE 3:**
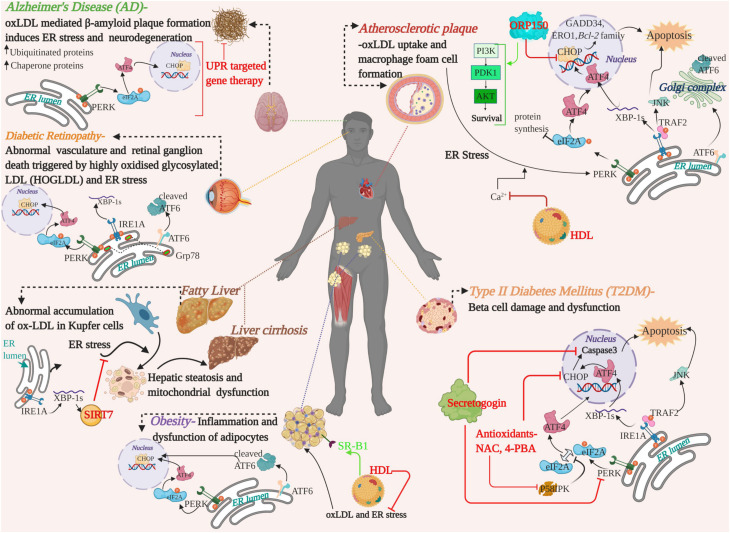
A graphical summary showing the pathological crosstalk between oxLDL and ER stress involved in the progression of various human diseases and therapeutic strategies developed on the basis of *in vitro*/*in vivo* studies in human or animal models. Therapeutic intervention of the targets and their mode of action are highlighted in red and green. oxLDL, oxidized LDL; ER, Endoplasmic Reticulum; UPR, Unfolded Protein Response; UPR arms, PERK, ATF6, IRE1A-, ORP150-oxysterol receptor protein 150; HDL, High Density Lipoprotein; NAC, N-Acetyl cysteine; 4-PBA, 4-phenyl butyric acid; SIRT7, Sirtuin 7; PI3K, Phosphoinositide 3-Kinase; PDK1, Phosphoinositide-dependent kinase-1; AKT/PKB, Protein Kinase B, serine/threonine kinase; SR-B1, scavenger receptor B1.

The mutual crosstalk between oxLDL and ER stress have been implicated in a range of emerging chronic diseases and widened the realms of our current understanding on the mechanism of dysfunction associated with these conditions. Figure 3 depicts a summary of the mechanisms and available therapeutic evidence. Apart from the curative potential of HDL, antioxidants or regulators of lipid metabolism; small molecules targeting the UPR or ER stress sensors and lncRNA/miRNA mediated modulation of oxLDL are also gaining interest. For instance, small molecule-based antagonist drugs such as 4-PBA or TUDCA against UPR component-mediated proteostatic impairment are gaining momentum. But the UPR also plays a substantial role in immune system regulation, cell differentiation and energy control (Todd et al., 2008; Dufey et al., 2014). For this reason, a key concern that arises with this approach are the underlying side-effects on the normal physiology (Gonzalez-Teuber et al., 2019). Targeting the altered phospholipids of oxLDL for active immunization with modified LDL derivatives or passive immunization with anti-oxLDL antibodies against atherosclerosis were successful in rabbit and murine models (Samson et al., 2012). In a similar line, anti-pneumococcal antibodies targeting pneumococci and mildly oxidized LDL greatly reduced atherosclerosis in mice, and the human trials are in progress, with no available data (Shekhar et al., 2018; Poznyak et al., 2021; Shiri-Sverdlov et al., 2021). Apoptosis signal-regulating kinase 1 (ASK-1) is upregulated by the IRE1A arm via TRAF2 phosphorylation and initiates the JNK pathway mediated apoptosis and fibrosis of the liver. The pharmacological administration of Selonsertib- an ASK-1 inhibitor, had promising effects in the second phase of trial in NASH patients. The results of the international phase three trials proved Selonsertib as a safe drug targeting ASK-1 but failed to reduce the progression of NASH (Loomba et al., 2018; Harrison et al., 2020). The detrimental convergence of oxidative stress with ER stress, wherein oxLDL serves as a linker that exacerbates the progression of the disease, is a double-edged sword and needs to be addressed in depth. In spite of the identification of serum oxLDL as the key risk factor in various diseases and its pharmacological intervention, an efficient translation of this therapeutic strategy from bench to bedside is yet to be achieved. Clinical challenges that limit the identification of serum oxLDL are the lack of pharmacokinetically and pharmacodynamically validated human antibodies against oxLDL as well as a high-throughput imaging system for the detection of oxLDL. Hence, advancements in these areas of research would provide a deeper understanding to adopt hybrid therapeutic strategies to combat the pathogenic players.

It is noteworthy to mention that abnormal accumulation of diacylglycerols (DAG), ceramides and other modified lipoproteins- in tissues or in circulation, also contribute to dyslipidemia and bring about irreversible organ dysfunction. Again, oxLDL-induced autophagy augments the severity of lipid-associated diseases. However, this review attempts to streamline the vast knowledge currently available on oxLDL and ER stress mediated dysfunction and identify common elements for a better perception of maladies affiliated to these key factors.

## Author Contributions

BA conceptualized the idea and critically reviewed the manuscript. DV wrote and edited the original draft and prepared the illustrations. Both authors contributed to the article and approved the submitted version.

## Conflict of Interest

The authors declare that the research was conducted in the absence of any commercial or financial relationships that could be construed as a potential conflict of interest.
